# Audit of physical health monitoring in a forensic psychiatric community caseload

**DOI:** 10.1192/bjo.2021.317

**Published:** 2021-06-18

**Authors:** Neeti Sud, Michael Lacey

**Affiliations:** Cumbria, Northumberland, Tyne And Wear NHS Foundation Trust

## Abstract

**Aims:**

Adherence to Cumbria Northumberland Tyne and Wear NHS Foundation (CNTW) Trust physical health monitoring guidelines for a caseload of community forensic psychiatry patients residing at Westbridge supported accommodation was audited to identify areas for improvement in practice. It was also our aim to highlight the delay in obtaining non-urgent investigations due to the need to minimize COVID infection transmission risks.

**Method:**

Data were collected from mental health and acute trust electronic records (Rio and ICE) of all patients taking antipsychotic medications currently care coordinated by the Westbridge Forensic Community Mental Health Team (FCMHT) between January 2020 and January 2021 (8 patients). Analysis of compliance with standards set by Trust guidelines was made.

**Result:**

In the chosen audit period, compliance with physical health monitoring standards was below target of 100% (80% compliance for bloods, 50% for ECG). Reasons for non-compliance were unexpected restrictions in service availability (e.g. temporary closure of walk-in ECG clinic) and one omission of sending a prolactin levels request.

**Conclusion:**

The need for practice adaptation and advance planning by team in anticipation of potential delays was identified. Request for routine bloods and ECGs will now be made two months before the annual due dates to compensate for delays in the new process with plan to continue re-audit yearly.

